# The pathogenesis of DLD-mediated cuproptosis induced spinal cord injury and its regulation on immune microenvironment

**DOI:** 10.3389/fncel.2023.1132015

**Published:** 2023-05-09

**Authors:** Chaochen Li, Chunshuai Wu, Chunyan Ji, Guanhua Xu, Jiajia Chen, Jinlong Zhang, Hongxiang Hong, Yang Liu, Zhiming Cui

**Affiliations:** ^1^The Affiliated Hospital 2 of Nantong University, Nantong University, The First People’s Hospital of Nantong, Nantong, China; ^2^Key Laboratory for Restoration Mechanism and Clinical Translation of Spinal Cord Injury, Nantong, China; ^3^Research Institute for Spine and Spinal Cord Disease of Nantong University, Nantong, China

**Keywords:** spinal cord injury (SCI), cuproptosis, immune microenvironment, DLD, machine learning

## Abstract

**Introduction:**

Spinal cord injury (SCI) is a severe central nervous system injury that leads to significant sensory and motor impairment. Copper, an essential trace element in the human body, plays a vital role in various biological functions and is strictly regulated by copper chaperones and transporters. Cuproptosis, a novel type of metal ion-induced cell death, is distinct from iron deprivation. Copper deprivation is closely associated with mitochondrial metabolism and mediated by protein fatty acid acylation.

**Methods:**

In this study, we investigated the effects of cuproptosis-related genes (CRGs) on disease progression and the immune microenvironment in acute spinal cord injury (ASCI) patients. We obtained the gene expression profiles of peripheral blood leukocytes from ASCI patients using the Gene Expression Omnibus (GEO) database. We performed differential gene analysis, constructed protein-protein interaction networks, conducted weighted gene co-expression network analysis (WGCNA), and built a risk model.

**Results:**

Our analysis revealed that dihydrolipoamide dehydrogenase (DLD), a regulator of copper toxicity, was significantly associated with ASCI, and DLD expression was significantly upregulated after ASCI. Furthermore, gene ontology (GO) enrichment analysis and gene set variation analysis (GSVA) showed abnormal activation of metabolism-related processes. Immune infiltration analysis indicated a significant decrease in T cell numbers in ASCI patients, while M2 macrophage numbers were significantly increased and positively correlated with DLD expression.

**Discussion:**

In summary, our study demonstrated that DLD affects the ASCI immune microenvironment by promoting copper toxicity, leading to increased peripheral M2 macrophage polarization and systemic immunosuppression. Thus, DLD has potential as a promising biomarker for ASCI, providing a foundation for future clinical interventions.

## Graphical Abstract

The graphical abstract is divided into two main sections, upper and lower. The upper section comprises three subsections, from left to right, which respectively represent the peripheral blood immune microenvironment in non-spinal cord injury, the involved biological processes and genes, and the peripheral blood immune microenvironment after spinal cord injury. The lower section also contains three subsections, from left to right, which respectively represent the legend for immune cells, the application of artificial intelligence in the field of spinal cord injury, and the specific trends of changes in the peripheral blood immune microenvironment after spinal cord injury. 
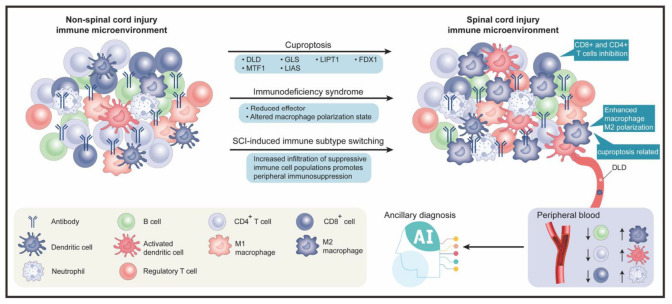

## 1. Introduction

Spinal cord injury (SCI) is commonly caused by external trauma and is considered as one of the most serious injuries in traumatology, requiring advanced experience, practice, and knowledge to ensure the best outcomes for patients (Fehlings and Nguyen, 2010; Huang et al., 2020). SCI affects 1.3 million people in North America alone, and the direct lifetime cost per patient ranges from $1.1 to $4.6 million (Wilson et al., 2013; Ahuja et al., 2016; J Spinal Cord Med, 2016). Furthermore, SCI-related disability and death rates have been increasing in recent years (Byra, 2016; Casper et al., 2018).

The severity of spinal cord injuries can be divided into two stages: primary and secondary. Primary-stage injury is defined as direct mechanical damage to tissue, typically due to shearing, tearing, acute stretching, or sudden acceleration and deceleration (Wilkerson and Hayes, 2010; Van Gassen et al., 2015). Secondary injuries can be categorized as acute (within 48 h), subacute (2–14 days), intermediate (14 days to 6 months), or chronic (over 6 months) (Badhiwala et al., 2019). Hemorrhage and disruption of the blood-spinal cord barrier (BSCB) expose the spinal cord to inflammatory cells, such as neutrophils, macrophages, and cytokines, accompanied by the release of cytotoxic byproducts (Kim et al., 2017). Edema progresses during the subacute phase, causing further vascular damage, calcium dysregulation, inflammation, and persistent ischemia, which cyclically promote the cytotoxic microenvironment (Singh et al., 2012; Wang et al., 2014; Liu et al., 2015; Khan et al., 2018). The intermediate and chronic phases of SCI are characterized by dynamic vascular remodeling, alterations in extracellular matrix composition, and reorganization of local and distal neural circuits (Bradbury and Burnside, 2019). Additionally, after SCI, peripheral lymphoid organs (e.g., the spleen) lose sympathetic innervation, resulting in SCI-induced immune deficiency syndrome (SCI-IDS), which substantially increases the risk of peripheral infection (Brommer et al., 2016). Peripheral infections are the leading cause of death in patients with spinal cord injury (Savic et al., 2017; Kriz et al., 2021). Moreover, infections and associated hyperthermia can further impair the function of the central nervous system (CNS) after an SCI (Carpenter et al., 2020). Unfortunately, no curative treatment options are available for improving neurological outcomes after SCI (Burns et al., 2017; Badhiwala et al., 2018).

Unlike treatments for primary injuries, those for traumatic SCI focus on minimizing secondary injuries (Lee et al., 2018), achieved through the use of methylprednisolone (MP) and early surgical decompression (Badhiwala et al., 2018). However, studies have shown that high-dose MP treatment in the early stages of ASCI does not result in better sensory recovery (*p*-value = 0.07), but instead causes gastrointestinal bleeding (*p*-value = 0.04) and respiratory infections (*p*-value = 0.04) (Liu et al., 2019). Therefore, high doses of MP should be used with caution as routine treatment for ASCI in the early stages. As the global population ages, cervical spine injuries account for an increasing proportion of traumatic spinal cord injury (Devivo, 2012). Researchers have found that patients with cervical acute spinal cord injury (ASIA) who undergo early surgery (within 24 h) show better functional recovery after six months than those who undergo late surgery (more than 24 h) (Fehlings et al., 2012). Nevertheless, early surgical intervention for ASCI may encounter numerous obstacles, including a lack of operating room availability, transporting patients from injury sites or other centers, a lack of specialized operating room teams, and a lack of on-call surgeons (Glennie et al., 2017). This means a large proportion of patients are likely to miss the optimal time to undergo surgery, depriving them of timely treatment and compromising their clinical outcomes. Due to limited sensitivity and specificity, the application of therapeutic strategies, such as pharmacotherapy (Kjell et al., 2015), cell therapy (Cofano et al., 2019), biomaterials (Zuidema et al., 2014), and functional electrical stimulation (Kapadia et al., 2014; Bonizzato et al., 2018), on a large clinical scale has been difficult. Recent studies suggest that the pathogenesis of SCI is closely related to the characteristics and dynamics of the infiltrating monocyte-derived macrophages (MDM) (Milich et al., 2019). As the field of SCI research grows, bioinformatics research based on next-generation sequencing attracts increasing attention. Gene expression analysis of three datasets (GSE92657, GSE93561, and GSE189070) in the GEO database identified a gene with high auxiliary value in SCI (Tong et al., 2022). Lai et al. (2013) investigated gene expression in thoracic intrinsic spinal cord neurons of 12 SCI rat models and 12 healthy control rats, identifying three genes of potential interest for future research (Lai et al., 2013). SCI-IDS can exacerbate peripheral infections after SCI, further impairing central nervous system function and resulting in increased mortality and complications. Therefore, studying changes in the immune microenvironment following SCI is necessary to further aid in the diagnosis of SCI and its adjuvant treatment.

Among essential trace elements, copper (Cu) plays an important role in growth, metabolism, and regulatory functions related to oxidative stress. It also potentially contributes to the pathophysiology of SCI and neural regeneration following injury (Che et al., 2016; Inesi, 2017; Guengerich, 2018; Timon-Gomez et al., 2018). Despite its usefulness as a cofactor for enzymes across the animal kingdom, copper can be toxic, resulting in cell death even at modest intracellular concentrations (Ge et al., 2022). The role of metal ions in SCI is diverse and important. A recent study shows that SCI patients exhibit a significant increase in iron deposits in their motor cortex, ultimately resulting in ferroptosis in motor neurons and impaired recovery of motor function (Feng et al., 2021). In a different study, zinc inhibited neuronal ferroptosis through the NRF2/HO-1 and GPX4 signaling pathways, exerting a neuroprotective effect (Ge et al., 2021). Following ferroptosis, a new metal ionic cell death pathway called cuproptosis has recently gained attention. It is associated with aggregation of fatty acylated proteins and proteotoxic stress resulting from excessive copper accumulation (Tsvetkov et al., 2022). Ten genes are implicated in the process of cuproptosis: FDX1, DLAT, LIPT1, PDHB, LIAS, DLD, and PDHA1 are positively regulated, while MTF1, GLS, and CDKN2A are negatively regulated (Tsvetkov et al., 2022). Cuproptosis has been shown to play a role in a variety of diseases. Wilson disease (WD) is associated with cuproptosis and is characterized by copper accumulation in cells and organs. Thus, copper chelators may be effective in treating WD (Aggarwal and Bhatt, 2018). A recent study evaluated the role of cuproptosis in hepatocellular carcinoma and identified a prognostic long non-coding (lnc) RNA profile associated with cuproptosis to predict response to immunotherapy (Zhang et al., 2022). Despite this, it remains unclear whether cuproptosis can be applicable as a therapeutic option for patients with spinal cord injuries. Serum copper levels have been found to be significantly higher in patients with spinal cord injuries than in healthy subjects (*p*-value = 0.002) (Salsabili et al., 2009). Abnormalities in serum copper levels after SCI could provide new insights into the pathogenesis of SCI.

Dihydrolipoamide dehydrogenase (DLD), a multifunctional oxidoreductase, is an essential component of multiple mitochondrial multienzyme complexes, and it is known to induce cuproptosis (Fan et al., 2014; Tsvetkov et al., 2022). Numerous studies have highlighted the importance of DLD in cell death. For example, DLD induced hyperphosphorylation of microtubule-associated tau protein, which led to neurodegeneration in Alzheimer’s disease (Ahmad, 2018). Moreover, DLD has been shown to produce a significant amount of reactive oxygen species (ROS) in melanoma cells, inducing apoptosis (Dayan et al., 2019). In addition, DLD is known to promote ferroptosis in head and neck cancers (Shin et al., 2020). Although cell death is inevitable during the acute phase of spinal cord injury, it is unknown whether DLD influences this process.

Our study aimed to evaluate the effects of cuproptosis-related genes (CRGs) on ASCI progression and the immune microenvironment using a multi-omics and multi-dimensional approach, in addition to identifying peripheral blood biomarkers for acute (A) SCI. Therefore, an exploratory examination of the relationship between DLD, ASCI disease progression, and changes in the immune microenvironment was performed using bioinformatics analysis, including differential expression analysis, protein–protein interaction (PPI) network analysis, centrality analysis, weighted gene co-expression network analysis (WGCNA), risk model construction, deep learning-based clinical prediction model construction, functional enrichment analysis, molecular subtype analysis, and immune infiltration analysis. Our findings suggested that DLD affects the peripheral immune microenvironment in ASCI and induces M2 polarization of macrophages, which exacerbates SCI-IDS and adversely impacts ASCI outcome. Moreover, DLD shows potential as a peripheral blood biomarker for ASCI. As a result of our analysis, it becomes clear that CRGs play an important role in ASCI development, as well as providing a basis for therapeutic applications of cuproptosis regulators in ASCI.

## 2. Materials and methods

### 2.1. Data sources and preprocessing

We downloaded single-array ASCI patients’ RNA-seqencing (seq) data from the GEO database^1^ to eliminate the possibility of multi-array batch effects interfering with our results. The criteria for gene chip selection were as follows: (1) Complete raw RNA-sequencing data were available, (2) RNA-sequencing of peripheral blood leukocytes from patients with spinal cord injuries, (3) Within 48 h after the injury, peripheral blood samples were collected from the patient, and (4) Complete clinical baseline information was available for the patients. The selected gene microarray dataset was GSE151371 (Kyritsis et al., 2021), with *Homo sapiens* as the selected species and peripheral blood leukocytes as the selected tissue type. The dataset included data from 20 control patients without spinal cord injuries (Control group) and 38 ASCI patients (ASCI group). Data from microarray experiments were normalized using the Bioconductor package limma (Phipson et al., 2016). Clinical data for ASCI patients were obtained from the GSE151371 dataset (Table 1). Missing values and outlier samples were removed before training neural networks.

**TABLE 1:** Baseline information.

Characteristic	Levels	Overall
n		58
Group, *n* (%)	SCI	38 (65.5%)
	Control	20 (34.5%)
Sex, *n* (%)	F	19 (32.8%)
	M	39 (67.2%)
Race, *n* (%)	Asian	4 (6.9%)
	Black or African-American	5 (8.6%)
	Hispanic	25 (43.1%)
	Other	3 (5.2%)
	Unknown	16 (27.6%)
	White	5 (8.6%)
Prior CNS pathology, *n* (%)	No	41 (70.7%)
	Yes	12 (20.7%)
	Unknown	5 (8.6%)
Concurrent TBI, *n* (%)	No	48 (82.8%)
	Yes	8 (13.8%)
	Unknown	2 (3.4%)
NLI grouped, *n* (%)	Cervical	18 (47.4%)
	Lumbar	2 (5.3%)
	Thoracic	10 (26.3%)
	Unknown	8 (21.1%)
ASIA impairment scale, *n* (%)	A	12 (31.6%)
	B	4 (10.5%)
	C	6 (15.8%)
	D	11 (28.9%)
	Unknown	5 (13.2%)
Age, median (IQR) 51 (39, 66)		
ISS, median (IQR) 21 (17, 32.5)		
Blood draw time, median (IQR) 22.5 (17, 40)		

### 2.2. Expression profiling of CRGs

To determine the expression of CRGs in ASCI, we identified the chromosomal locations using the R software package RCircos (Zhang et al., 2013). Next, we analyzed the expression of CRGs in the ASCI and control groups using the Wilcoxon test and visualized the results using the ggplot2 (Ito and Murphy, 2013) package. The differential expression of CRGs was analyzed using the R package limma and visualized using the ggplot2 package. Since small changes in the nervous system can have a substantial impact, we set a cutoff at a *p*-value of <0.05 to ensure the comprehensiveness of the differential expression analysis.

Next, to analyze the correlation and interaction between positive (FDX1, DLAT, LIPT1, PDHB, LIAS, DLD, and PDHA1) and negative (MTF1, GLS, and CDKN2A) cuproptosis regulators, we performed Spearman correlation analysis on CRGs and visualized the results using the R package ggcorrplot.

### 2.3. Risk model and clinical prediction model construction

We first identified key genes associated with ASCI using univariate logistic regression analysis, and then used the least absolute shrinkage and selection operator (LASSO) algorithm to confirm the correlation between key genes and ASCI. Furthermore, a variance inflation factor cutoff of four was used to exclude multicollinearity in multivariate logistic regression analysis of ASCI. To validate the results of the multivariate logistic regression and assist in the diagnosis of ASCI, we developed a nomogram prediction model based on these results. A calibration curve was used to evaluate the performance of the nomogram model in identifying patients with ASCI.

After considering the ASIA score as the dependent variable, we incorporated the ASCI-related potential biomarker (DLD) and eight clinical features into the model. The clinical features included sex, age, race, prior central nervous system pathology, injury severe score, concurrent traumatic brain injury, blood draw time, and damaged spinal cord stage. We used the R package keras to model the classifier architecture using neural networks. The ASIA scores were compressed according to grades with the A, B, and C levels designated as level 2, whereas the D and E levels were designated as level 1. This neural network consisted of two layers, in which the input layer activation function was “relu” and the output layer activation function was “sigmoid,” combined with a rmsprop optimizer and a custom penalty function. The ASCI patients were randomly assigned to training and validation sets in a 3:7 ratio. To measure the performance of the clinical prediction model, receiver operating characteristic (ROC) curves were calculated by comparing predicted and observed values from the neural network.

### 2.4. CRGs functional enrichment analysis and gene set variation analysis

CRG annotation using GO and KEGG pathway enrichment analysis was performed using the clusterProfiler package (Yu et al., 2012) in R. Statistical significance was determined using a false discovery rate of 0.05.

To determine whether there were differences between different groups regarding biological processes, we performed gene set variation analysis (GSVA) using the gene expression profiling dataset of ASCI patients. Gene set enrichment analysis (GSEA) evaluates whether two biological states are significantly different from each other by comparing a gene set (Subramanian et al., 2005). GSVA is a sub-algorithm of GSEA that estimates changes in pathway and biological process activity in samples of expression datasets. Using the annotation catalog (msigdb.v7.4.symbols.gmt) from the MSigDB database (Liberzon et al., 2015), we performed GSVA using the R package GSVA (Hanzelmann et al., 2013), and employed linear fitting and Bayesian network algorithms to determine the differences between the ASCI and control groups in the relevant GSVA pathways. A *p*-value threshold of 0.05 was used to determine statistical significance.

### 2.5. Weighted gene co-expression network analysis (WGCNA)

Using the R package WGCNA (Langfelder and Horvath, 2008), we performed a WGCNA analysis of the eigengene set in ASCI. First, to eliminate outliers from the standardized gene expression data, we performed hierarchical clustering. In the next step, we adopted the *R*^2^ and slope values to determine the optimal soft threshold, as well as to validate the scale-free network. A dissimilarity analysis, with a threshold of 0.25, was used to determine the adjacency matrix and topology matrix, and dynamic shear trees were analyzed to identify network modules (deepSplit = 2, miniClusterSize = 30). Finally, we performed the correlation analysis in conjunction with the ASCI phenotype data.

### 2.6. Construction of protein–protein interaction (PPI) network

The PPI network of CRGs was constructed using the STRING database, and the sub-networks were extracted using the MCODE plugin of Cytoscape software (version: 3.9.1) (Shannon et al., 2003). Next, we examined the centrality of the PPI network of CRGs in four dimensions including betweenness centrality, closeness centrality, degree centrality, and stress centrality. Moreover, we analyzed the co-expression of ASCI-related WGCNA module genes, differentially expressed CRGs, PPI sub-network genes of CRGs, and the results of multivariate logistic regression.

### 2.7. Identification and correlation analysis of immune infiltration in ASCI

The extent of immune cell infiltration in the control and ASCI groups was calculated using the single-sample Gene Set Enrichment Analysis (ssGSEA) algorithm. According to gene expression data, ssGSEA can be used to determine the population of immune cells within a sample (Subramanian et al., 2005). CIBERSORT is an analytical tool developed by Newman et al. to estimate the abundance of different cell types in mixed cell populations by using gene expression data (Newman et al., 2019). We validated immune infiltration results using the CIBERSORT algorithm in R. Statistically significant differences in immune cell proportions between normal and diseased sample groups were calculated using the Wilcoxon test, with a *p-value* of 0.05 being considered statistically significant. To determine the stability of the immune infiltration analysis results, based on the R package immunedeconv and ImmuCellAI, we also used different algorithms for validation, including: quanTIseq, Xcell, MCP-counter, and ImmuCellAI (Sturm et al., 2019; Miao et al., 2020).

Our analysis of ASCI key genes and immune characteristics was conducted using the R package corrr to quantify the correlation between ASCI key genes and immune characteristics. Based on the ASIA score, we divided the ASCI patients into two groups: the ASIA-high group, which included levels A, B, and C, and the ASIA-low group, which included levels D and E. Next, we further explored the effect of an altered immune microenvironment on ASCI disease progression through differential expression and correlation analysis.

### 2.8. Construction of the ASCI-related molecular subtype

To analyze the potential subtypes among the CRGs of ASCI patients, we first organized the expression matrix of CRGs, then compressed the data distribution using normalization, and finally performed consensus clustering analysis using the hclust algorithm (sample resampling ratio: 80%, number of resamplings: 1,000, maximum number of clusters: 7). The R package pheatmap was used to visualize molecular typing results. To evaluate ASCI molecular typing, we used the T-distributed Stochastic Neighbor Embedding (tSNE) algorithm. According to the results of the analysis of ASCI-related molecular subtypes, Spearman’s coefficient was used to determine the relationships between key genes and ASCI-related molecular subtypes.

### 2.9. qRT-PCR and ELISA experiment

White cells were extracted from peripheral blood through Peripheral Blood Leukocyte Isolation Kit (TBD, WBC1077K), strictly following the manufacturer’s instructions. We collected peripheral blood (5 ml) from patients in the ASCI group (*n* = 3) and control group (*n* = 3) using anticoagulant tubes. Carefully suck the blood sample with a straw and add it to the liquid level of the separation solution (5 ml), then centrifuge (500–800 *g*, 30 min). After centrifugation, carefully aspirate all circular milky white cell layers (one or two layers) using a pipette, add them to 10 ml of cleaning solution, and mix the cells evenly. Then centrifuge at low speed (250 *g*, 10 min). Repeat washing 2–3 times to obtain the required white blood cells. qRT-PCR and ELISA experimental processes are similar to our previous studies (Wu et al., 2022). The primers in qRT-PCR are LDHD-F sequence (5’–3’ agc ctg agc acc gtg tta cc), LDHD-R Sequence (5’–3’ gcc agg aca gga tgc gta gg), which purchased from Sangon Biotech (Shanghai, 007177). The Human DLD ELISA Kit was purchased from ZCi Biotech (Shanghai, ZC-56138). Measure the absorbance (OD value) using an enzyme-linked immunosorbent assay at a wavelength of 450 nm. Both qRT-PCR and ELISA experiments were repeated three times.

### 2.10. Statistical analysis

Data processing and analysis were conducted using the R software (version 4.2.0). When comparing two continuous variable groups, an independent Student’s *t*-test was used to estimate the statistical significance of normally distributed variables, and Mann–Whitney U tests were used to examine differences between non-normally distributed variables. Statistical significance of the two categorical variable groups was determined using the chi-square or Fisher’s exact test. Spearman correlation analysis was used to measure the correlation coefficients of different genes. The statistical *p*-values were two-tailed, and statistical significance was set at *p* < 0.05.

## 3. Results

### 3.1. Gene chip quality control

**FIGURE 1:**
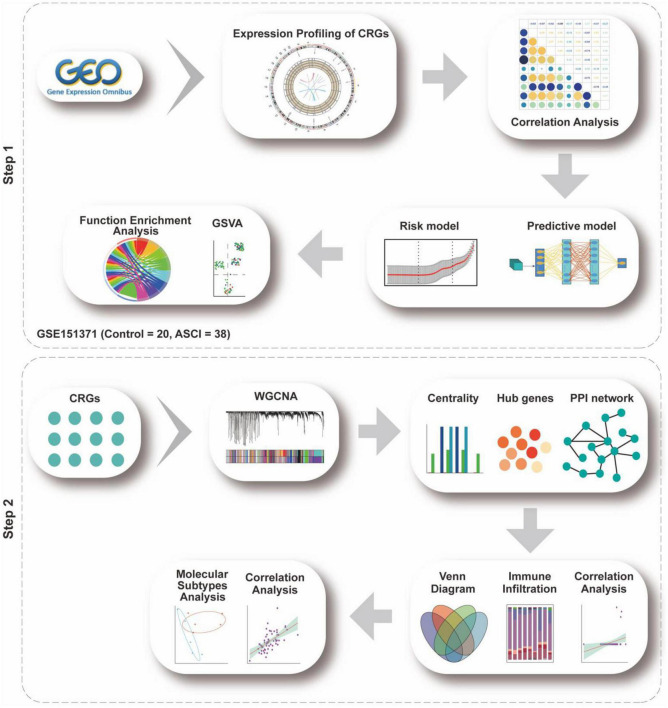
Analysis flow chart. The study flow chart is divided into two main sections according to the study sequence. Each icon represents schematically an analysis or a collection of data to be analyzed.

The flow chart for this study can be found in Figure 1.

We analyzed the expression levels in ASCI and control groups from the GEO dataset using background calibration to evaluate how ASCI patients express CRGs overall (Supplementary Figure 1).

### 3.2. Expression profile of CRGs in ASCI

**FIGURE 2:**
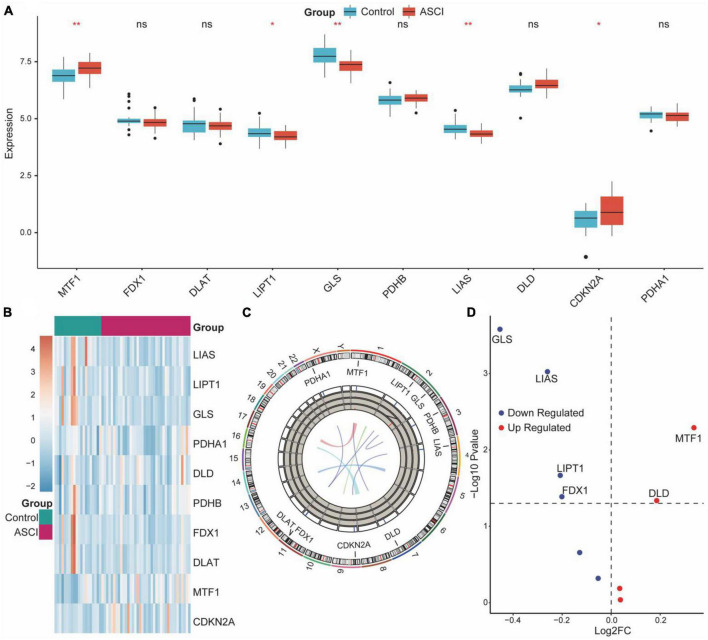
Overall expression of cuproptosis-related genes (CRGs) in acute spinal cord injury (ASCI) patients. **(A)** Differences in the expression of CRGs between the ASCI and control groups; ns indicates *p*-value = 0.05, * indicates *p*-value < 0.05, and ** indicates *p*-value < 0.01. **(B)** Expression heat map of CRGs. **(C)** Chromosome localization map of CRGs. **(D)** Volcano plot of the results of the differential genetic analysis of CRGs.

CRG expression distribution and chromosomal localization were analyzed to determine the overall expression of CRGs in ASCI patients (Figures 2B, C). Next, we determined whether the expression of CRGs in the ASCI group differed from that in the control group using the Wilcoxon test. The results demonstrated that MTF1, LIPT1, GLS, LIAS, and CDKN2A were significantly differentially expressed (Figure 2A). Subsequently, we performed differential gene expression analysis for CRGs in ASCI patients. We found that DLD and MTF1 were significantly upregulated in ASCI patients, whereas GLS, LIAS, LIPT1, and FDX1 were significantly downregulated in ASCI patients (*p*-value < 0.05) (Figure 2D). To ensure the comprehensiveness of the study, we combined the results from Wilcoxon’s test and differential gene expression analysis.

**FIGURE 3:**
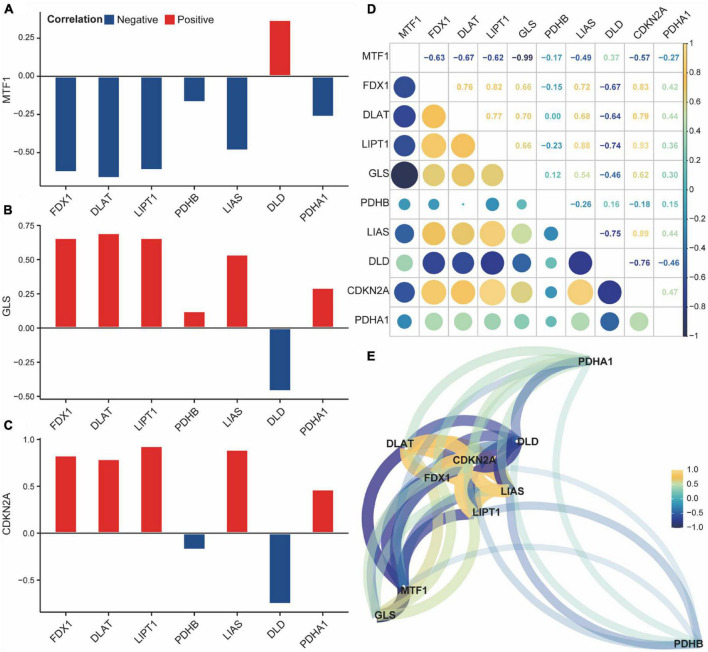
Correlation of cuproptosis-related genes (CRGs) in acute spinal cord injury (ASCI) patients. **(A–C)** Correlation histogram plot of CRGs; the abscissa represents positive cuproptosis regulators, and the ordinate represents negative cuproptosis regulators; Colour code indicates correlation. **(D)** Correlation heat map of CRGs; numbers represent correlation coefficients. **(E)** Correlation network diagram of CRGs; numbers represent correlation coefficients.

Next, we analyzed the correlation and interaction between positive and negative regulators within CRGs using the Spearman algorithm (Figure 3E). There was a positive correlation between DLD and MTF1, and a negative correlation between DLD, GLS, and CDKN2A (Figures 3A–C). Genes with a correlation coefficient greater than 0.7 were considered significantly correlated, and a significant negative correlation was observed between DLD and CDKN2A (Figure 3D).

### 3.3. Construction of risk models

**FIGURE 4:**
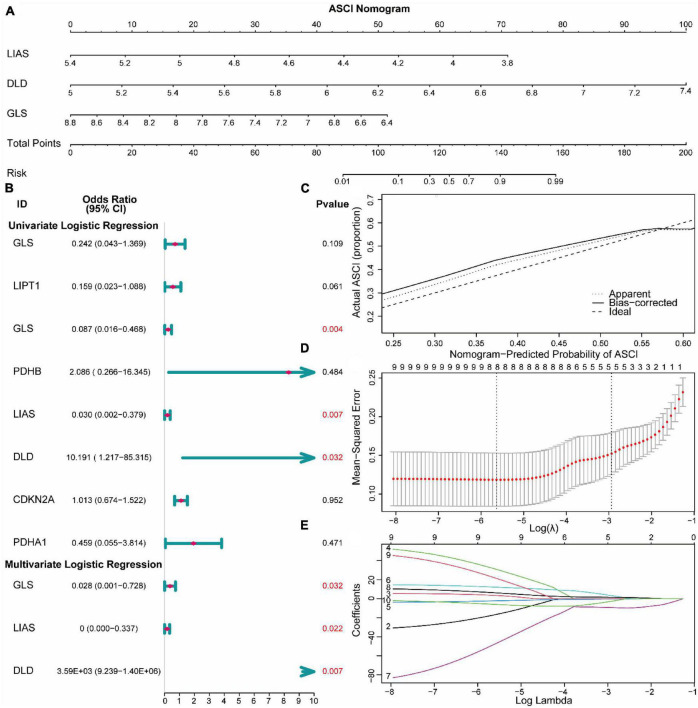
Construction of risk models for acute spinal cord injury (ASCI). **(A)** Nomogram prediction model for ASCI. **(B)** Forest plot base on logistic regression. **(C)** Calibration curves for the Nomogram prediction model. **(D,E)** LASSO regression recognized essential genes of ASCI.

To analyze the expression of CRGs in ASCI, we first performed a univariate logistic regression analysis to identify key genes associated with ASCI. We employed the LASSO algorithm to narrow down the analysis and validate the key genes associated with ASCI (Figures 4B, D, E). In a multivariate logistic regression model, ASCI-related eigengenes were incorporated, and GLS, LIAS, and DLD were identified as independent risk factors for ASCI (*p*-value < 0.05) (Figure 4B). Based on the multivariate logistic regression results, a predictive nomogram was constructed to predict the risk of ASCI (Figure 4A). The calibration curve demonstrated that the nomogram prediction model, based on the independent risk factors of ASCI, could identify patients with ASCI with excellent accuracy (Figure 4C).

### 3.4. Construction of clinical prediction models

**FIGURE 5:**
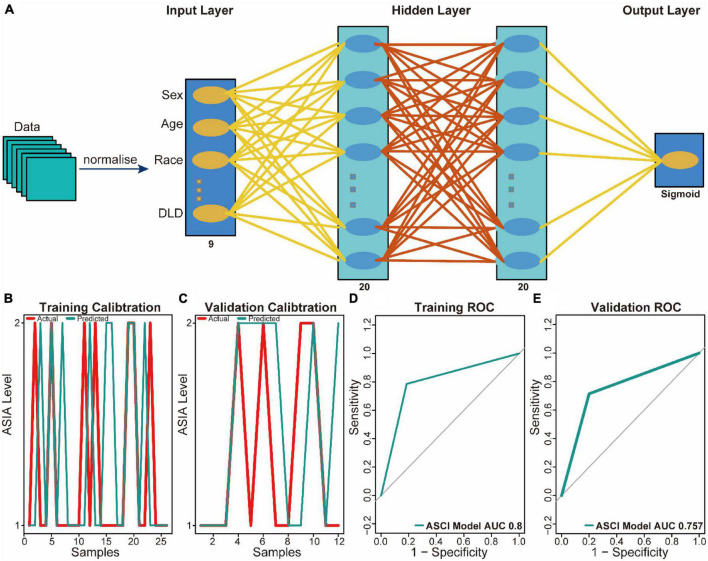
Predictive power analysis of neural network model for acute spinal cord injury (ASCI). **(A)** Architecture diagram of neural network model. **(B)** Calibration curve for the neural network model in the training set; the abscissa represents patients, and the ordinate represents the levels of ASIA. **(C)** Calibration curve for the neural network model in the validation set; the abscissa represents patients and ordinate represents the levels of ASIA. **(D)** Receiver operating characteristic (ROC) curve of the training set of the neural network model; the abscissa represents the specificity and ordinate represents the sensitivity. **(E)** ROC curve of the validation set of the neural network model; the abscissa represents the specificity and ordinate represents the sensitivity.

To aid clinical diagnosis and treatment, we integrated DLD expression levels and clinical characteristics into our model, constructing an architecture of back-propagation neural networks with classifiers (Figure 5A). Based on the calibration curve, the model demonstrated excellent classification performance in both training and validation datasets (Figures 5B, C). A comparison between predicted and actual ASCI values was conducted to evaluate the performance of the neural network, resulting in ROC curves with area under the curve (AUC) of 0.8 and 0.757 in the training set and validation set, respectively (Figures 5D, E). These results confirm the outstanding ability of the neural network clinical prediction model to predict neurological function in ASCI patients. Moreover, by using the custom penalty function, we enhanced the goodness of fit of the neural network model for small datasets, thereby preventing the occurrence of overfitting.

### 3.5. Functional enrichment analysis

**FIGURE 6:**
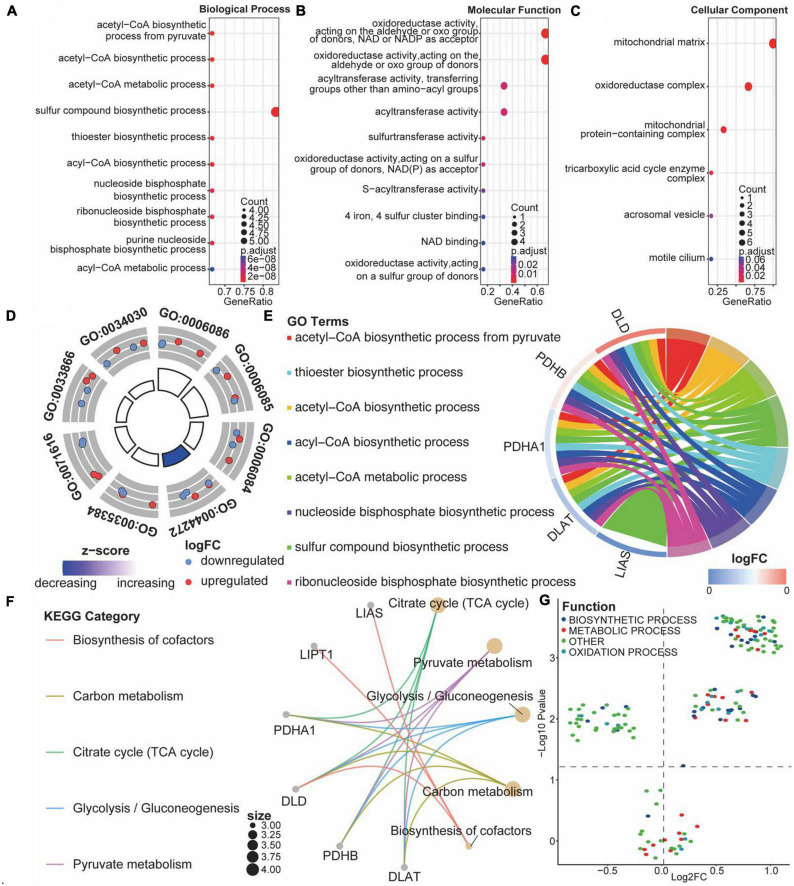
Functional enrichment analysis of cuproptosis-related genes (CRGs). **(A–C)** Bubble diagram of the first 10 biological processes, molecular functions, and cellular components items, with horizontal coordinates indicating GeneRatio, vertical coordinates indicating gene ontology (GO) terms, dot size indicating the number of genes, and dot color indicating adjust *p-value*. **(D)** Circle diagram of the first eight biological processes items, with the outer circle dot color representing upregulated and downregulated genes and the inner circle color representing activation or repression of GO terms. **(E)** String diagram of the first eight biological processes, the left outer half-circle represents genes within the pathways, the color indicates log fold change (FC), the right outer half-circle color indicates GO terms, and the inner connecting line indicates the association of GO terms with genes. **(F)** Interaction network diagram of the Kyoto encyclopedia of genes and genomes (KEGG) pathways. **(G)** Volcano plot of differential expression of gene set variation analysis (GSVA); colors represent different biological process.

Comparing ASCI and control patients, we analyzed the effects of CRGs on biologically relevant functions (Table 2). The GO functional annotation results of CRGs revealed that biological processes were dominated by differentially expressed genes, such as the biosynthesis of acetyl-CoA from pyruvate, acetyl-CoA metabolic process, and acetyl-CoA biosynthetic process (Figure 6A); molecular functions such as antioxidant activity, acting on the aldehyde or oxo group of donors, NAD or NADP as acceptor, acyltransferase activity, and sulfurtransferase activity (Figure 6B); and cellular components such as mitochondria matrix, oxidoreductase complex, and mitochondrial protein-containing complex (Figure 6C). The first eight enrichment results of the GO biological process examined the regulation of CRGs (Figures 6D, E). The next step was to conduct an interactive study of enrichment of CRGs in KEGG pathways, and the results indicated that these CRGs were enriched in pathways such as the citrate cycle (TCA cycle), pyruvate metabolism, and glycolysis/gluconeogenesis (Figure 6F).

**TABLE 2:** Function enrichment analysis.

Category	ID	Description	*p*-value
BP	GO:0006086	Acetyl-CoA biosynthetic process from pyruvate	9.66E-13
BP	GO:0006085	Acetyl-CoA biosynthetic process	8.96E-12
BP	GO:0006084	Acetyl-CoA metabolic process	1.53E-10
BP	GO:0044272	Sulfur compound biosynthetic process	1.72E-10
BP	GO:0035384	Thioester biosynthetic process	4.35E-10
MP	GO:0016620	Oxidoreductase activity, acting on the aldehyde or oxo group of donors, NAD or NADP as acceptor	2.08E-10
MP	GO:0016903	Oxidoreductase activity, acting on the aldehyde or oxo group of donors	4.28E-10
MP	GO:0016747	Acyltransferase activity, transferring groups other than amino-acyl groups	2.11E-03
MP	GO:0016746	Acyltransferase activity	2.67E-03
MP	GO:0016783	Sulfurtransferase activity	3.26E-03
CC	GO:0005759	Mitochondrial matrix	2.12E-10
CC	GO:1990204	Oxidoreductase complex	1.52E-08
CC	GO:0098798	Mitochondrial protein-containing complex	2.71E-03
CC	GO:0045239	Tricarboxylic acid cycle enzyme complex	3.98E-03
CC	GO:0001669	Acrosomal vesicle	3.66E-02
KEGG	hsa00020	Citrate cycle (TCA cycle)	2.23E-09
KEGG	hsa00620	Pyruvate metabolism	1.45E-08
KEGG	hsa00010	Glycolysis/Gluconeogenesis	6.20E-08
KEGG	hsa01200	Carbon metabolism	5.54E-07
KEGG	hsa01240	Biosynthesis of cofactors	1.25E-04

We performed GSVA analysis with CRGs to verify the accuracy of GO and KEGG enrichment analyses. Biological processes were found to differ significantly from one another: biosynthetic process, metabolic process, and oxidation process were significantly upregulated after ASCI (Figure 6G). Based on these findings, CRGs may play a role in metabolic processes associated with SCI.

### 3.6. Weighted gene co-expression network analysis (WGCNA)

**FIGURE 7:**
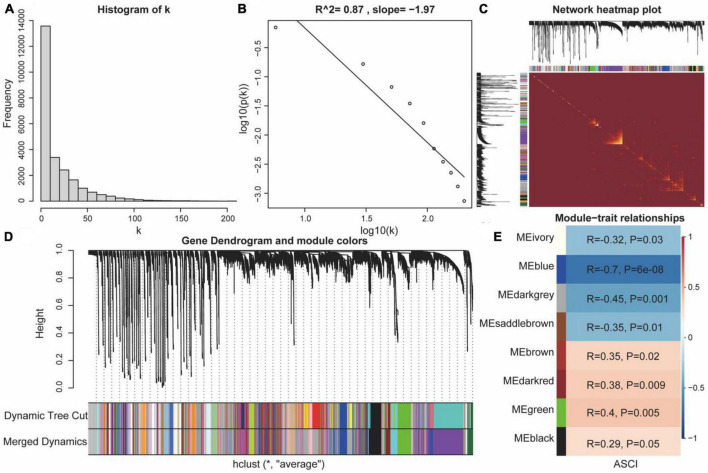
Weighted gene co-expression network analysis (WGCNA). **(A,B)** Scale-free network verification graph (*R*^2^ > 0.8, slope < 0), conforming to the scale-free network standard. **(C)** TOM network clustering heatmap. **(D)** Dynamic clipping tree clustering diagram; the abscissa is the clustering module and ordinate is the tree height. **(E)** Heat map of correlations between WGCNA network modules and acute spinal cord injury (ASCI).

To analyze the eigengene set of ASCI, we performed a WGCNA using all genes from the GSE151371 chip. First, we performed a hierarchical clustering analysis on the samples; then, outlier samples were removed, and a dynamic clipping tree was used to identify the network modules (Figure 7D). The scale-free network was verified upon selecting the best soft threshold. The results showed that R = 0.87 and slope = −1.97, and the scale-free network had been successfully established (Figures 7A–C). After excluding the gray module, we performed a correlation analysis using the scale-free network module and the external module (ASCI), revealing a significant relationship between the ivory, blue, darkgray, saddlebrown, brown, darkred, green, and black modules and ASCI (*p*-value < 0.05) (Figure 7E).

### 3.7. PPI network of CRGs between ASCI and control groups

**FIGURE 8:**
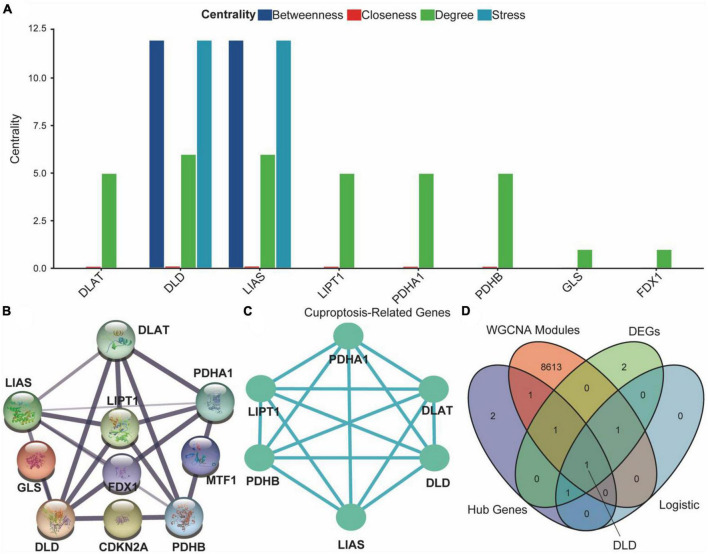
Protein–protein interaction (PPI) network of cuproptosis-related genes (CRGs). **(A)** Analysis of degree centrality, betweenness centrality, closeness centrality and stress centrality in PPI network. **(B)** PPI network of the CRGs; the width and colors of edges indicate the credibility of the evidence. **(C)** Network diagram of hub genes. **(D)** Venn diagram of hub genes, differential expression CRGs, acute spinal cord injury (ASCI)-related weighted gene co-expression network analysis (WGCNA) modules, and results of the multivariate logistic regression.

We explored differences in PPI networks by extracting protein interaction networks of CRGs from ASCI and control groups. The PPI network of CRGs was constructed using the STRING database, containing 17 interaction relationships and ten CRGs, with a confidence index of 0.7, an average local clustering coefficient of 0.733, and an enrichment *p*-value of 1.11e-16 (Figure 8B). Next, we used the Cytoscape software to extract the functional interaction subnets of the PPI network (Figure 8C). We also analyzed the centrality of PPI network nodes in four dimensions: betweenness centrality, closeness centrality, degree centrality, and stress centrality. The results indicated that DLD and LIAS occupy critical positions within the PPI network (Figure 8A). After conducting co-expression analyses of all CRGs, ASCI-related WGCNA modules, differentially expressed CRGs, and multivariate logistic regression, we found that DLD was not only highly correlated with ASCI but also exhibited significant changes in expression levels after ASCI (Figure 8D). Thus, cuproptosis regulator DLD may play an important role in ASCI, which was the focus of our next analysis.

### 3.8. Immune infiltration and correlation in ASCI and control groups

**FIGURE 9:**
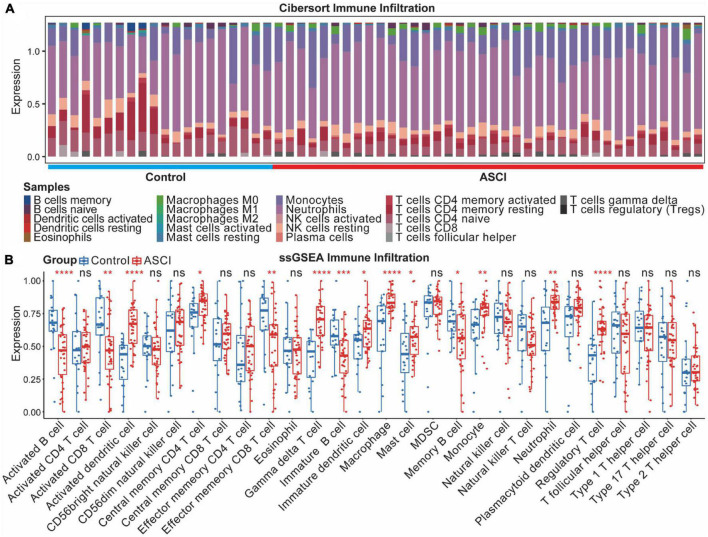
Acute spinal cord injury (ASCI) immune infiltration analysis. **(A)** Differential expression of infiltrating immune cells between ASCI and control groups determined using CIBERSORT immune infiltration analysis. **(B)** Differential expression of immune infiltrating cells in the ASCI and control groups based on single-sample gene set enrichment analysis (ssGSEA). ns indicates *p*-value = 0.05, * indicates *p*-value < 0.05, ** indicates *p*-value < 0.01, *** indicates *p*-value < 0.001, and **** indicates *p*-value < 0.0001.

ASCI patients were assessed using the CIBERSORT algorithm for their immune profile and level of immune cell infiltration (Figure 9A). To further elucidate changes in the immune microenvironment of ASCI patients, we estimated the extent of immune cell infiltration using ssGSEA (Figure 9B). After ASCI, the number of activated B cells and activated CD8 T cells significantly decreased, while the number of macrophages significantly increased (*p*-value < 0.05). To evaluate the stability of the immune infiltration analysis, we used several immune infiltration algorithms for validation and obtained similar conclusions (Supplementary Figures 2A–D).

**FIGURE 10:**
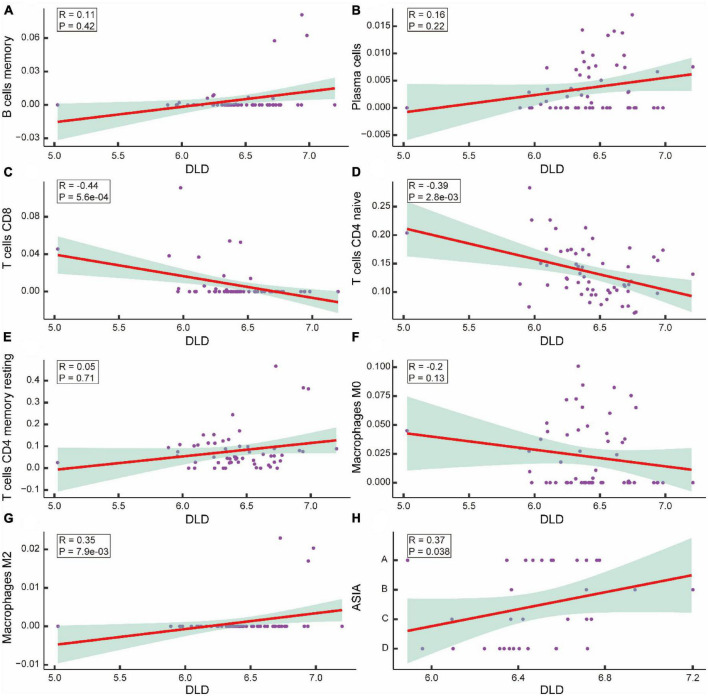
Correlation analysis of acute spinal cord injury (ASCI) key genes and immune infiltrating cells. **(A–G)** Scatter plot of the correlation between DLD and immune infiltrating cells, including B cells memory, Plasma cells, T cells CD8, T cells CD4 naive, T cells CD4 memory resting, macrophage M0, and macrophage M2; R represents correlation coefficient, and *P* represents *p*-value. **(H)** Scatter plot of the correlation between DLD and ASIA levels; R represents correlation coefficient, and *P* represents *p*-value.

To determine whether CRGs play a role in the altered immune environment after ASCI, we analyzed the correlation between DLD and differentially expressed T cells, B cells, plasma cells, and macrophages. Correlations were considered significant when the p-value was less than 0.05. The results indicated that DLD and CD8 T cells were significantly negatively correlated (*R* = −0.44, *p* = 5.6e-04) (Figure 10C), DLD and CD4 naive T cells were significantly negatively correlated (*R* = −0.39, *p* = 2.8e-0.3) (Figure 10D), and DLD and M2 macrophages were significantly positively correlated (*R* = 0.35, *p* = 7.9e-03) (Figure 10G). There was no significant correlation between DLD and the remaining immune infiltrating cell population (*p*-value > 0.05) (Figures 10A, B, E, F). A significant positive correlation was also found between DLD and ASIA grades (*R* = 0.37, *p* = 0.038) (Figure 10H). We analyzed M2 macrophage levels in ASCI-high and ASCI-low patients to understand the role of immune cell abnormalities in ASCI disease progression. The results showed that the ASIA-high group had a significantly higher content of macrophage M2 (*p*-value < 0.05) (Supplementary Figure 2F). Moreover, M2 macrophage numbers and ASIA levels showed a significant positive correlation (*R* = 0.12, *p* = 0.046) (Supplementary Figure 2E). This suggests that peripheral blood macrophages M2 play an important role in the ASCI process.

### 3.9. Construction and correlation analysis of relevant molecular subtypes of ASCI

**FIGURE 11:**
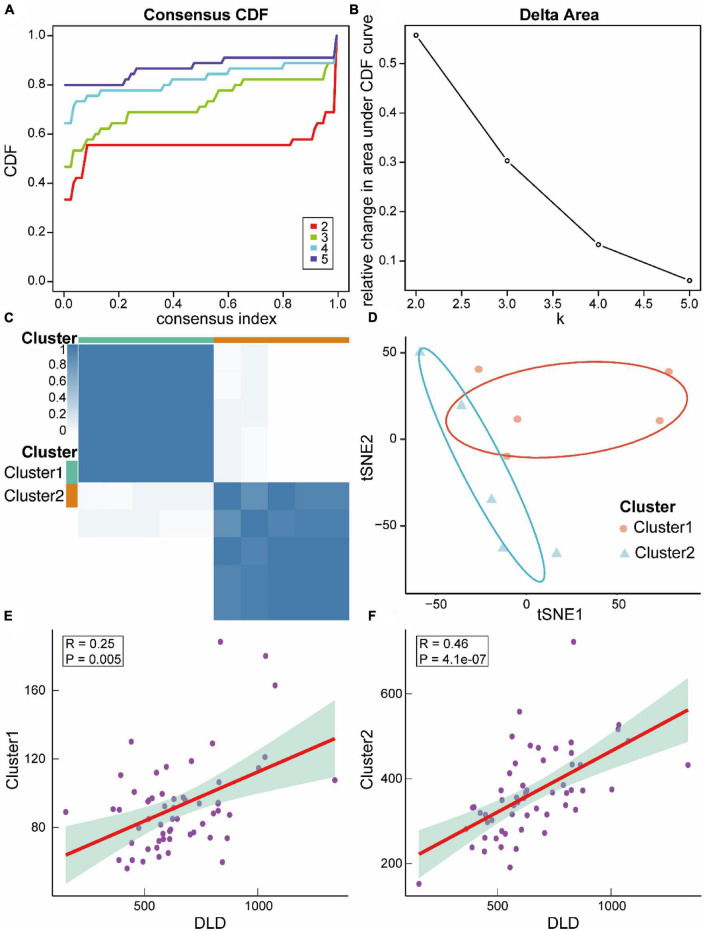
Relevant molecular subtypes and correlations of acute spinal cord injury (ASCI). **(A)** Cumulative distribution function (CDF) curve of consensus clustering of ASCI-related molecules; the abscissa represents the consensus index, and the ordinate represents the CDF index. **(B)** Relative change in the area under the CDF curve; the results show that it is divided into two types, and the change in trend is the most stable. **(C)** Cluster heat map of ASCI-associated molecular subtypes. **(D)** T-distributed Stochastic Neighbor Embedding (tSNE) analysis plot of ASCI-related molecular subtypes. **(E)** Scatter plot of the correlation between DLD and ASCI-related molecular subtype Cluster1; R represents correlation coefficient, and P represents *p*-value. **(F)** Scatter plot of the correlation between DLD and ASCI-related molecular subtype Cluster2; R represents correlation coefficient, and P represents *p*-value.

We then constructed ASCI subtypes based on their molecular characteristics. Based on the cumulative distribution function (CDF), it was optimal to have two subtypes, named Cluster1 and Cluster2 (Figures 11A–C). To verify the effect of ASCI molecular typing, tSNE analysis was performed. The results indicated that Cluster1 and Cluster2 have excellent resolution (Figure 11D). Finally, we calculated the association between the ASCI key gene DLD and the two ASCI molecular subtypes, finding that both Cluster1 (R = 0.25, *p* = 0.005) and Cluster2 (R = 0.46, *p* = 4.1e-07) were significantly positively correlated with DLD (Figures 11E, F).

### 3.10. qRT-PCR and ELISA experiment confirmed the increase expression of DLD in white cells after ASCI

**FIGURE 12:**
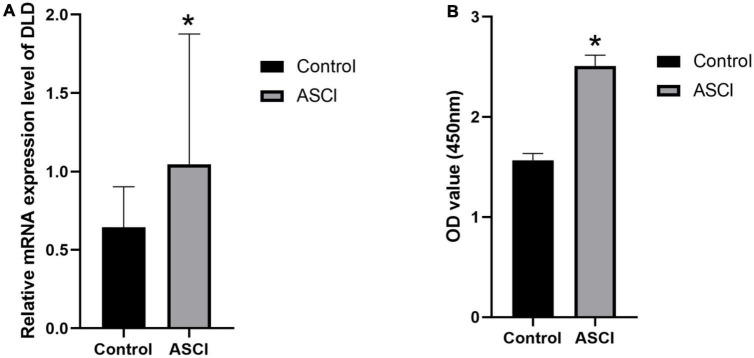
DLD expression in white cells from peripheral blood. **(A)** The mRNA (DLD) expression significantly increased in ASCI group in qRT-PCR experiment. **(B)** ELISA experiment detected a significant increase in DLD expression of ASCI group. (*p < 0.05).

To verify the expression changes of DLD after ASCI, we extracted peripheral white blood cells from patients in the experimental group and control group. qRT-PCR and ELISA experiment indicated that DLD was significantly increased in mRNA and protein level in white blood cells after ASCI (Figures 12A, B). These results suggested that DLD might be related to immune infiltration in peripheral white blood cells after ASCI.

## 4. Discussion

An unintentional spinal cord injury is an extremely serious condition, with dire consequences for the patient’s health and a significant financial burden (Wu et al., 2021). ASCI has been extensively studied, but no effective molecular targeted therapies have been validated. A new cell death pathway, cuproptosis, has recently gained increased attention (Tsvetkov et al., 2022). In the current study, we utilized human peripheral blood leukocyte data obtained from the GEO database to analyze the effects of CRGs on the ASCI immune microenvironment at a multi-omics level and their clinical significance. Using differential gene analysis, WGCNA, risk model construction, and PPI network centrality analysis, we identified potential ASCI peripheral blood diagnostic markers and the potential therapeutic target DLD. We integrated these results with clinical information from ASCI patients to construct a predictive model for ASCI. Based on our findings, the enriched biological processes and pathways in ASCI are closely related to metabolic and oxidative processes. The immune infiltration results indicated that systemic immune function was suppressed following ASCI. We constructed two molecular subtypes of ASCI and elucidated the role of CRGs in both subtypes. In the ASCI subtypes Cluster1 and Cluster2, the cuproptosis process, positively regulated by DLD, affected the peripheral immune microenvironment and induced the polarization of M2 macrophages, further exacerbating systemic immunosuppression following ASCI and affected the prognosis.

Numerous studies have highlighted the importance of immune cells in ASCI. For example, a high-segment spinal cord injury can be treated by targeting the spleen in order to regain immune homeostasis (Noble et al., 2018). The function of natural killer (NK) cells is affected following SCI, and thus, NK cells are considered potential therapeutic targets for SCI (Laginha et al., 2016). Consequently, in this study, we selected human peripheral blood leukocyte sequencing data and used only one chip to eliminate batch effects. Initially, we normalized the GSE151371 microarray data to identify the chromosomal location of CRGs. Among the six CRGs identified by differential gene analysis, DLD and MTF1 were significantly upregulated in ASCI patients. Accordingly, DLD and MTF1 are likely key genes involved in cuproptosis affecting ASCI. Next, to investigate whether dynamic regulation of the cuproptosis process occurs after ASCI, we analyzed the interaction between positive and negative cuproptosis regulators, and the results indicated that DLD was significantly negatively correlated with CDKN2A. However, univariate logistic regression indicated no correlation between CDKN2A and ASCI; thus, DLD may represent an independent risk factor for ASCI. Due to its role as a multifunctional oxidoreductase, DLD is involved in various processes, such as DNA binding, apoptosis mediation, and reactive oxygen species generation (Fernandez and Bolanos, 2016; Ambrus and dam-Vizi, 2018; Lin et al., 2019). It has been reported that inhibition of DLD counteracts oxidative stress in type 2 diabetes (Yang et al., 2019). Furthermore, another study showed that DLD inhibition reduces ischemic stroke damage through reduced oxidative stress, reduced cell death, increased Nrf2 signaling, and increased NQO1 activity (Wu et al., 2017a). Our results reveal similar evidence for the important role played by DLD in the metabolic and oxidative processes of ASCI.

Using the PPI network identified in this study, we discovered genes that play an important role in ASCI, specifically DLD and LIAS. Both univariate and multivariate logistic regressions analyses indicated that DLD, LIAS, and GLS were independent risk factors for ASCI. Based on the logistic regression results, we constructed a clinical prediction nomogram model. Using DLD, LIAS, and GLS expression levels, the model can accurately diagnose ASCI. A scale-free network using WGCNA was constructed to ensure the study was as comprehensive and stable as possible. Eight gene modules associated with ASCI were identified using this network. Next, we identified potential ASCI biomarkers from four aspects, including: linear relationships (logistic regression), scale-free networks (WGCNA), protein interaction relationships (PPI networks), and differential genes. Across all four latitudes, DLD showed significant importance. Therefore, DLD was confirmed to play an important role as an independent risk factor in the development of ASCI. After combining the clinical data of ASCI patients with the expression levels of DLD, we built a back-propagation neural network model that can be used to predict the neurological function of ASCI patients and assist with diagnosis and treatment. Using a custom penalty function, we optimized the fit of a neural network for a small training set, which not only achieved a higher recall rate, but also prevented under- or over-fitting. The clinical application value of DLD as a biomarker in the diagnosis and treatment of ASCI was further elucidated by the neural network clinical prediction model. In the enrichment analysis, the metabolism-related pathways such as pyruvate metabolism and citrate cycle (TCA cycle) were significantly enriched, suggesting that DLD played a critical role in the metabolic process of various diseases (Landgraf et al., 2017; Wu et al., 2017b; Purroy et al., 2020). After ASCI, metabolic and oxidative processes were active, as indicated by the results of the GO and GSVA analyses. Metabolism plays a significant role in ASCI, as dysregulated metabolic pathways are involved in pathological processes leading to tissue damage and functional impairment (Lepoutre et al., 2017; Neural Regen Res, 2021). Targeting metabolic pathways is a promising strategy for ASCI treatment, improving neuronal survival, promoting axonal regeneration, and reducing pathological processes (Meesters et al., 2017; Meier et al., 2017). Enriched metabolic pathways in ASCI include the TCA cycle, glycolysis, and ketone body metabolism. Their dysregulation leads to oxidative stress, cell death, and tissue damage, suggesting therapeutic potential for metabolic interventions (Lepoutre et al., 2017; Meier et al., 2017). Metabolic reprogramming may serve as a hallmark of ASCI progression and a potential target for therapy (Meier et al., 2017). Targeting metabolic pathways may offer new avenues for treating ASCI. Further research is needed to elucidate the underlying mechanisms and potential therapeutic benefits of metabolic interventions.

Compared to the control group, the proportions of mature B cells and mature CD8 T cells in the ASCI group decreased, indicating suppressed peripheral immune function. On the other hand, M2 macrophage levels were elevated in ASCI patients, indicating altered polarization tendencies among peripheral macrophages. Macrophage polarization is unstable and may be affected by various factors (Boutilier and Elsawa, 2021). The cuproptosis-related gene DLD has been implicated in immunity by several studies. Pseudomonas aeruginosa virulence may be influenced by DLD, which is a complement-regulatory protein-binding protein (Hallstrom et al., 2015). Another study indicated that DLD was an autoantibody target in patients with endometrial cancer (Yoneyama et al., 2014). Consequently, we propose a hypothesis that DLD following ASCI facilitates copper binding to lipidated components of the TCA cycle in peripheral blood, promoting copper-induced cell death. As a result, the immune microenvironment is disrupted, and macrophage polarization is altered, exacerbating SCI-IDS and ultimately worsening ASCI. Further analysis revealed that M2 macrophage was highly expressed in ASCI-high patients, adversely affecting their prognosis. ASIA grading and M2 macrophage levels were significantly positively correlated with DLD expression levels in ASCI patients. Consequently, high levels of DLD after ASCI likely contribute to macrophage polarization and a poor prognosis for ASCI patients. Several studies have demonstrated that M2 macrophage secretes suppressive cytokines to downregulate the immune response, a process that further exacerbates SCI-IDS (Ivashkiv, 2013; Murray et al., 2014). Thus, we can conclude that our hypothesis is correct. In addition, we found that DLD has potential as a therapeutic target for ASCI. A previous study identified three clinical phenotypes with different risks of death among hyperchloremia patients, allowing for precise treatment based on the patient’s clinical characteristics (Thongprayoon et al., 2021a). Thongprayoon et al. (2021b) divided hospitalized patients with acute kidney injury into four groups with different mortality risks, based on which adjuvant therapies were available (Thongprayoon et al., 2021b). To determine the applicability of DLD as a potential therapeutic target for ASCI, we identified two molecular subtypes associated with cuproptosis in ASCI patients and calculated the correlation between DLD expression levels and the two ASCI subtypes (Cluster1 and Cluster2). We found that DLD was significantly positively correlated with both ASCI subtypes Cluster1 and Cluster2, indicating that DLD may be a therapeutic target for ASCI in general.

Our study has multiple strengths. This is the first study to describe the effects of CRGs on the immune microenvironment of ASCI patients. Research on the immune microenvironment and cell death is an important component of SCI research, and programmed cell death is considered a key process in post-SCI recovery (Chavan et al., 2017; Prüss et al., 2017; Shi et al., 2021). Cuproptosis is a novel type of mitochondrial respiration-dependent metal ion cell death that differs from ferroptosis (Tsvetkov et al., 2022). It may be possible to develop new treatments for spinal cord injury due to this unusual mechanism. Furthermore, in contrast to most diagnostic models that focus primarily on ASCI disease states, our work also considers neurological function. The majority of current clinical prediction models use linear architecture but do not take the interference of multicollinearity into consideration. We used the variance inflation factor to eliminate the problem of multicollinearity in logistic regressions. Additionally, we used a more flexible neural network model to predict neurological function in ASCI patients, which not only had a high recall rate and a high goodness of fit but also could be continually evolved.

However, our study also has some limitations. First, this research relies heavily on bioinformatics analysis, and it needs more validation through both animal experiments and clinical trials. Second, in the case of human peripheral blood leukocyte gene chips, the volume of collected tissue samples is relatively small. Third, the generalizability of the clinical diagnostic model lacks external validation from patients at other medical centers. Although the clinical prediction model constructed in this study showed reasonable robustness (AUC of 0.757 for the validation set ROC curve), the detection ability of the deep learning model must be improved through the integration of a large amount of data. Finally, due to the heterogeneity of ASCI, not all ASCI patients exhibit significant peripheral immunosuppression, and further subgroup analyses should consider additional immune profiles of ASCI patients.

## 5. Conclusion

In conclusion, our study highlights DLD’s potential as a biomarker and therapeutic target for ASCI, and its association with M2 macrophage levels affecting patient prognosis. We developed a clinical prediction model and a neural network model for better diagnosis and treatment. Despite limitations, our findings emphasize the significance of metabolic pathways and the immune microenvironment in ASCI, encouraging further research on this topic.

## Data availability statement

The original contributions presented in this study are included in the article/Supplementary material, further inquiries can be directed to the corresponding author.

## Author contributions

CL, CW, and JC helped in the conception and design, data acquisition, analysis and interpretation, and critical revision of the article and final approval. GX, JC, JZ, HH, and YL helped in the data acquisition and final approval. ZC helped in the data acquisition, analysis, and drafting and critical revision of the article and obtained final approval. All authors have approved the submitted version of the manuscript.

## Footnotes

1
https://www.ncbi.nlm.nih.gov/geo/
