# Supplementary material for: The pathogenesis of DLD-mediated cuproptosis induced spinal cord injury and its regulation on immune microenvironment
Source: Front Cell Neurosci. 2023 May 9;17:1132015. doi: 10.3389/fncel.2023.1132015 (PMC10203164; doi:10.3389/fncel.2023.1132015)
Supplement: Supplementary file 1 [file Data_Sheet_1.docx]

Supplementary Material 01


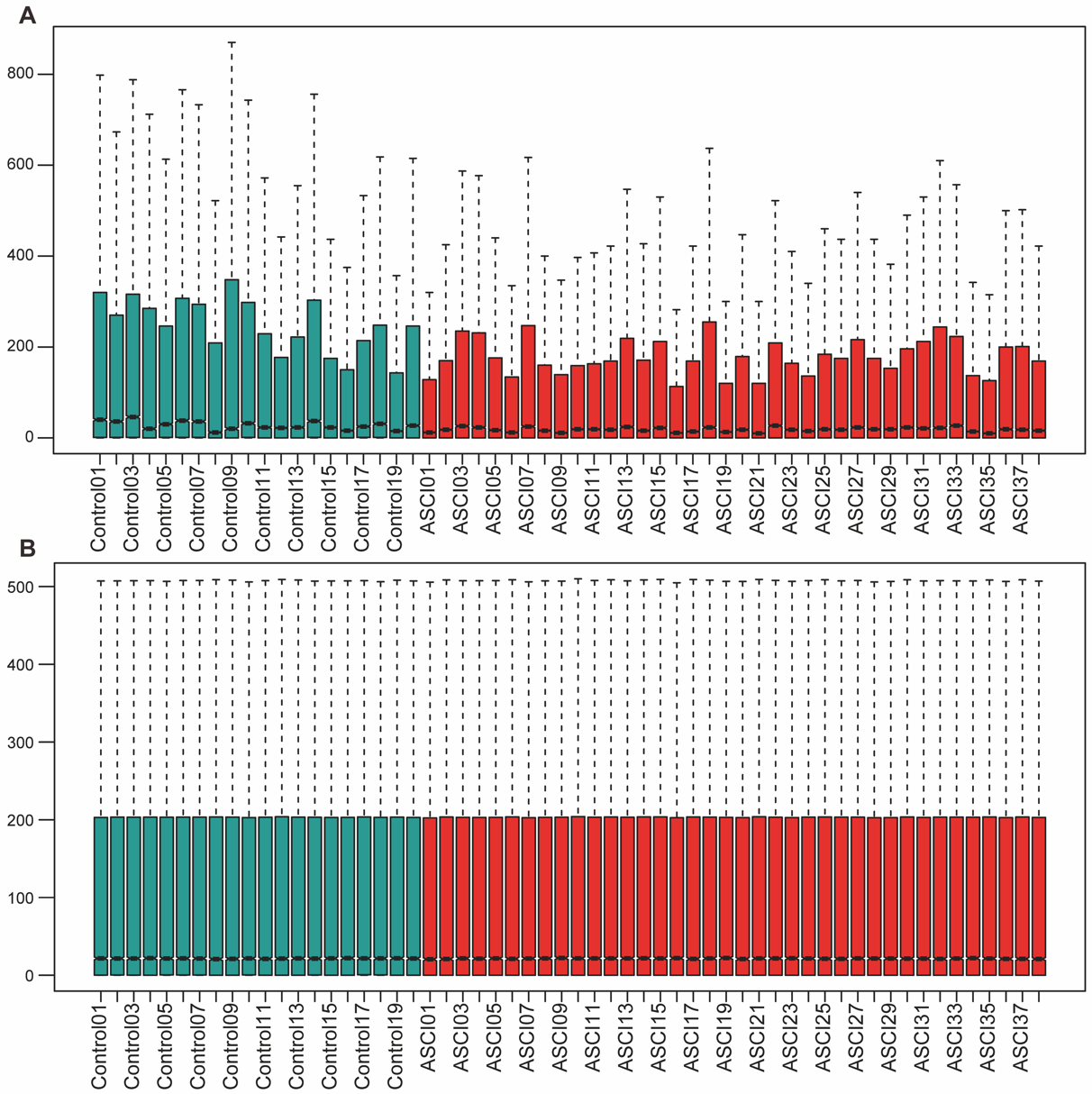


**Supplementary Figure S1.** Adjusted for acute spinal cord injury (ASCI) data. **(A)** GSE151371 chip data before correction. **(B)** GSE151371 chip data after correction.

**
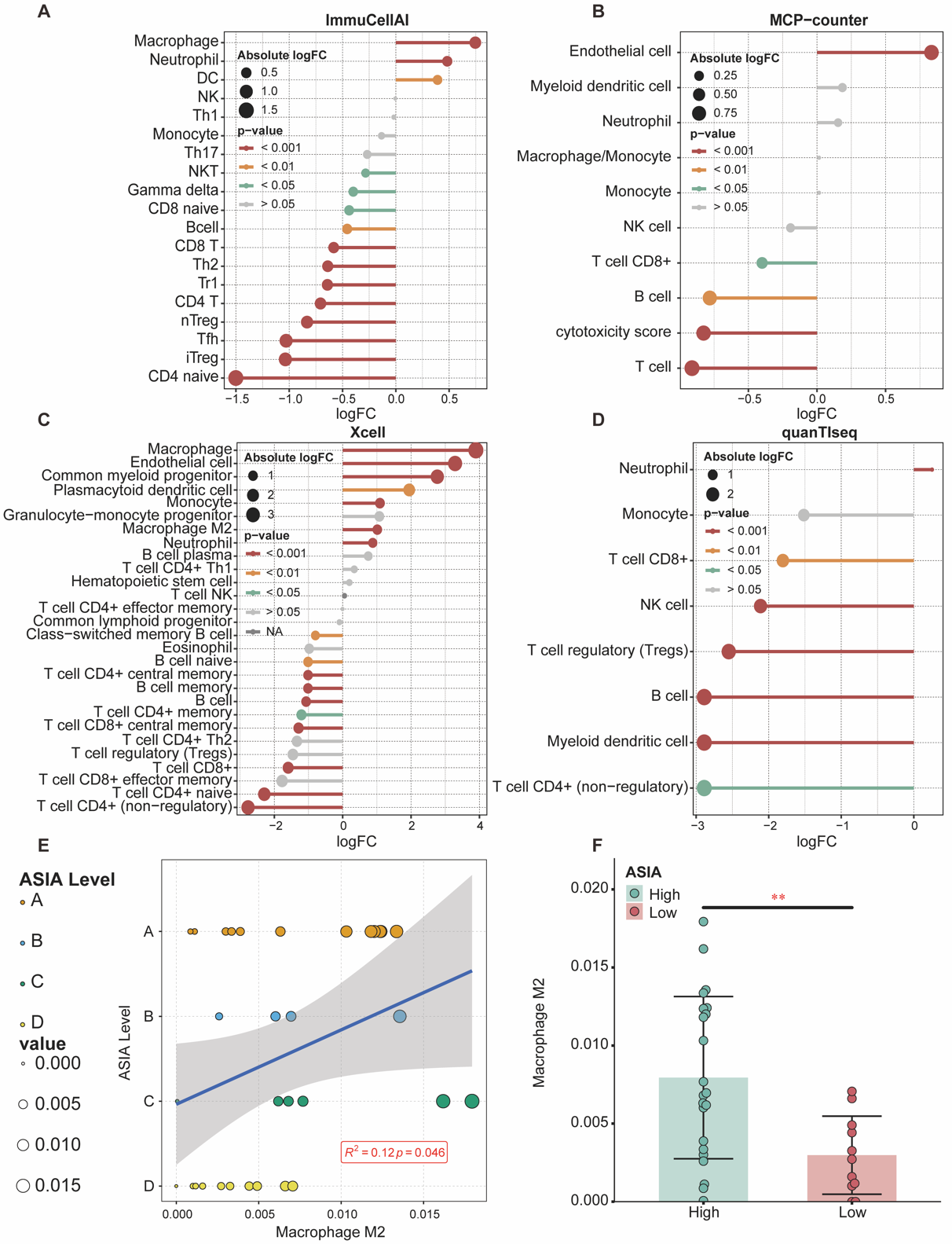
**

**Supplementary Figure S2.** Validation of the stability of immune infiltration analysis and correlation analysis. **(A-D)** Immune infiltration analysis lollipop plot of ImmuCellAI, MCP-counter, Xcell, and quanTIseq; the abscissa represents log fold change (logFC), the ordinate represents the immune cells, colors represent p-value, and the size of the dots represents absolute logFC. **(E)** Scatter plot of the correlation between macrophages M2 and ASIA levels; R represents correlation coefficient, P represents p-value, colors represent ASIA levels, and the size of the dots represents immune infiltration values. **(F)** Differential expression of macrophages M2 in the ASIA-high and ASIA-low groups; the abscissa represents immune infiltration values of macrophages M2, the ordinate and colors represent the ASIA-high and ASIA-low groups, and ** represents p-value < 0.01.
